# Pre-Clinical Models of Heart Failure with Preserved Ejection Fraction: Advancing Knowledge for Device Based Therapies

**DOI:** 10.1007/s10439-025-03821-z

**Published:** 2025-08-25

**Authors:** Nina Langer, Andreas Escher, Caglar Ozturk, Andrew F. Stephens, Ellen T. Roche, Marcus Granegger, David M. Kaye, Shaun D. Gregory

**Affiliations:** 1 Department of Mechanical and Aerospace Engineering, Monash University, Melbourne, VIC Australia; 2 Victorian Heart Institute, Victorian Heart Hospital, Melbourne, VIC Australia; 3 Institute for Medical Engineering and Science, Massachusetts Institute of Technology, Cambridge, MA USA; 4 Bioengineering Science Research Group, School of Engineering, University of Southampton, Southampton, UK; 5 School of Electrical Engineering and Robotics, Queensland University of Technology, Brisbane, QLD Australia; 6 Department of Cardiac Surgery, Medical University of Vienna, Vienna, Austria; 7 The Department of Cardiology, The Alfred Hospital, Melbourne, VIC Australia; 8 Centre for Biomedical Technologies and School of Mechanical, Medical, and Process Engineering, Queensland University of Technology, Brisbane, QLD Australia

**Keywords:** HFpEF, Mock circulation loops, In-vitro models, In-silico models, Ex-vivo models, In-vivo models

## Abstract

Heart failure with preserved ejection fraction (HFpEF) is a growing health problem worldwide, accounting for half of all heart failure cases. HFpEF patients present with diverse underlying causes and symptoms, making diagnosis and treatment challenging. Current pharmacological therapies are inadequate, while approved device-based therapies have shown limited success due to patient heterogeneity. This underscores the need for improved pre-clinical models, critical for guiding the design and development of effective therapeutic devices. This paper presents an overview of current pre-clinical HFpEF models, including in-silico, in-vitro, ex-vivo, and in-vivo approaches, aimed at advancing the understanding of HFpEF physiology and the development of device-based therapies. We examined each model's ability to replicate key HFpEF characteristics, discuss their respective strengths and limitations, and highlight their role in supporting the creation of clinically relevant solutions. Additionally, the potential of emerging advancements is explored.

## Introduction

Heart failure, affecting 64 million people worldwide [1], is the leading cause of death globally. Approximately half of all cases are heart failure with preserved ejection fraction (HFpEF), a condition characterized by stiff ventricles and normal ejection fraction. HFpEF contrasts with heart failure with reduced ejection fraction (HFrEF), which involves weakened myocardium and dilated ventricles [2, 3]. The prevalence of HFpEF is rising, placing an increasing burden on healthcare systems [4, 5]. Despite a five-year mortality rate of 47% and poorer long-term outcomes compared to many cancers [6, 7], HFpEF remains poorly understood and inconsistently defined [8]–[10]. Current classifications vary, using underlying mechanisms, hypertension status, or comprehensive clinical characteristics [11]–[13].

While HFrEF has well-established pharmaceutical and device-based treatments, HFpEF treatment options are still evolving [14, 15]. Sodium-glucose co-transporter 2 (SGLT2) inhibitors have emerged as the primary pharmaceutical treatment shown to provide benefits across most HFpEF populations and are widely recommended as initial therapy [16]. However, despite these advancements, many HFpEF patients remain symptomatic, highlighting the critical need for alternative treatment strategies, particularly device-based interventions [17]. Emerging devices target specific pathophysiological changes in HFpEF: inter-atrial and atrium to coronary sinus shunts aim to reduce left atrial pressure; left ventricular expanders enhance filling capacity and stroke volume; and mechanical circulatory support devices decompress the left atrium and may increase cardiac output. These devices, ranging from early development to clinical trials, represent a critical frontier in HFpEF treatment [15].

Certain device-based approaches may be effective for specific patient populations but not for others, as demonstrated by the REDUCE LAP-HF II trial for the Interatrial Shunt Device (IASD) [18]. Additionally, clinical trials are expensive, making thorough preclinical evaluation essential for ensuring success. As a result, preclinical HFpEF models are crucial in refining and advancing these therapies before clinical implementation. By replicating HFpEF's distinct anatomical and hemodynamic features, they facilitate the development, evaluation, optimization and personalization of device-based treatments. Pre-clinical models support understanding of HFpEF pathophysiology [19]–[21], improving device safety and efficacy [11, 22]–[24], and informing personalized clinical decision-making like placement strategies and intervention timing [25, 26]. .

A variety of HFpEF models have been developed, but there is little synergy between them, and no comprehensive clear guidance on their appropriate use, timing, or applications. Furthermore, there is no comprehensive summary outlining their benefits and limitations to highlight their current capabilities for device development and the gaps that remain. This paper aims to bridge that gap by providing a review and analysis of existing HFpEF preclinical models while outlining the necessary steps to establish a comprehensive suite of evaluation tools.

## Scope

This review examines pre-clinical HFpEF models, evaluating their ability to replicate disease characteristics, their limitations, and their potential to advance personalized and device-based therapies for this underserved patient population. By analyzing the strengths and weaknesses of these models, we highlight their critical role in driving innovative HFpEF treatment strategies and discuss potential future enhancements to improve their clinical relevance.

A systematic review of pre-clinical HFpEF models was conducted, focusing on studies published between 1996 and 2024. Literature searches in PubMed, Google Scholar, and Scopus identified studies using keywords such as “HFpEF preclinical model”, “HFpEF pre-clinical model,” “HFpEF computational model”, “HFpEF mock circulation loop”, “HFpEF animal model” and “HFpEF device testing” combined lead to 1346 results. After excluding duplicates, and initial screening of titles and abstracts, only studies relevant to device testing and hemodynamic evaluation were included, excluding those focused solely on drug development narrowing the selection to 32 publications. For models utilized in multiple studies, the earliest publication was prioritized. By focusing on models tailored for device evaluation, this work aims to inform and inspire future advancements in device-based HFpEF research.

## State-of-the-Art Models in HFpEF Research

### Model Overview

**Fig. 1:**
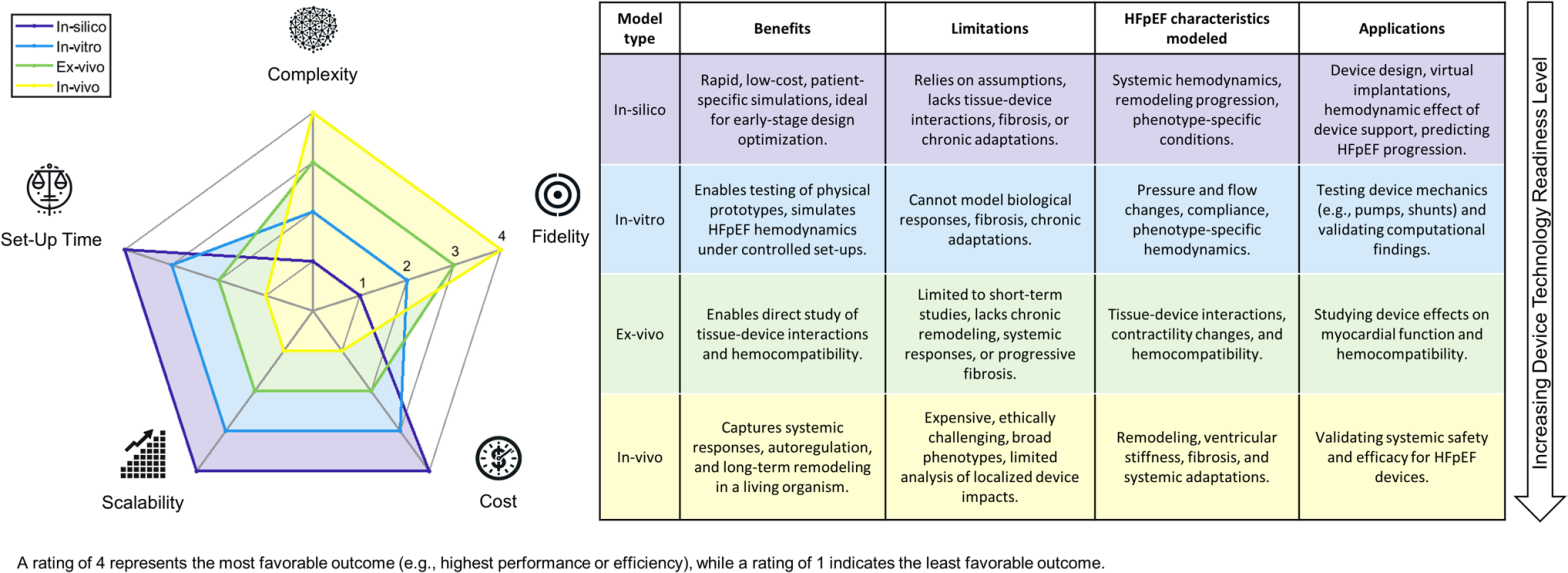
Radar graph comparing in-silico, in-vitro, ex-vivo and in-vivo models regarding complexity, fidelity, cost efficiency, scalability and set-up time (left) and tabular comparison (right). The complexity of in-silico and in-vitro models can vary from a simple set-up for initial evaluation, to highly complex assemblies with numerous components and increased fidelity. For visual simplicity, the graph in Figure 1 refers to the simplest method of each. This figure is the author’s own work, and does not comprehensively cover all models, nor do all models always fit within their category. Contains graphics created with AI.

State-of-the-art HFpEF models can be divided into in-silico, in-vitro, ex-vivo and in-vivo models. Each of those categories provides inherent benefits and shortcomings. Figure 1 shows a radar graph comparing in-silico, in-vitro, ex-vivo and in-vivo models regarding complexity, fidelity, cost, scalability and set-up time.

In-silico studies provide high adaptability, scalability, and control to assess surgical fit [27, 28] and device interaction with the cardiovascular system [11, 20, 23, 24, 29], aiding refinement of device design, performance, and placement. Hemodynamic responses to device-based treatments can be simulated using lower-dimension models or more detailed higher-dimensional approaches for local flow, hemocompatibility, and structural analysis. However, lower-dimension models involve many simplifications, and both approaches still face limitations in predicting biological responses such as cardiac remodeling.

In-vitro models physically model the biomechanics and hemodynamics of HFpEF patients [24, 27, 30]–[33]. However, to comprehensively model the cardiovascular system including hemodynamic interactions and adaptations, a number of components including autoregulatory responses are required, resulting in a complex set-up [28, 30, 34, 35]. Further, remodeling is typically not replicated as most of the materials used are non-biological and non-adaptive.

Ex-vivo models enable realistic biological responses and direct observation of tissue changes, supporting analysis of cardiac mechanics and hemodynamics [34]. They are especially useful for studying device interaction with a beating heart, offering insight into mechano-energetics and biocompatibility. However, biocompatibility and hemocompatibility are often poorly assessed, as setups using blood typically use simplified, non-physiological conditions. These models also face limitations: they are hard to tailor to specific phenotypes, may not reflect the intended disease state, and mainly capture acute effects, missing longer-term adaptive responses like neural or hormonal regulation.

In-vivo animal models are commonly used in drug and device development, offering insight into pressures and flows in major vessels under realistic physiological conditions [36]–[38]. They are effective for evaluating structural changes and blood compatibility. However, replicating HFpEF conditions remains challenging, and measuring in less accessible anatomical regions is difficult. Additionally, these studies are resource-intensive and costly.

The following section will discuss existing HFpEF models within each category, and highlight the HFpEF characteristics each model can replicate in addition to the applications of the models.

### In-Silico Models

**Fig. 2:**
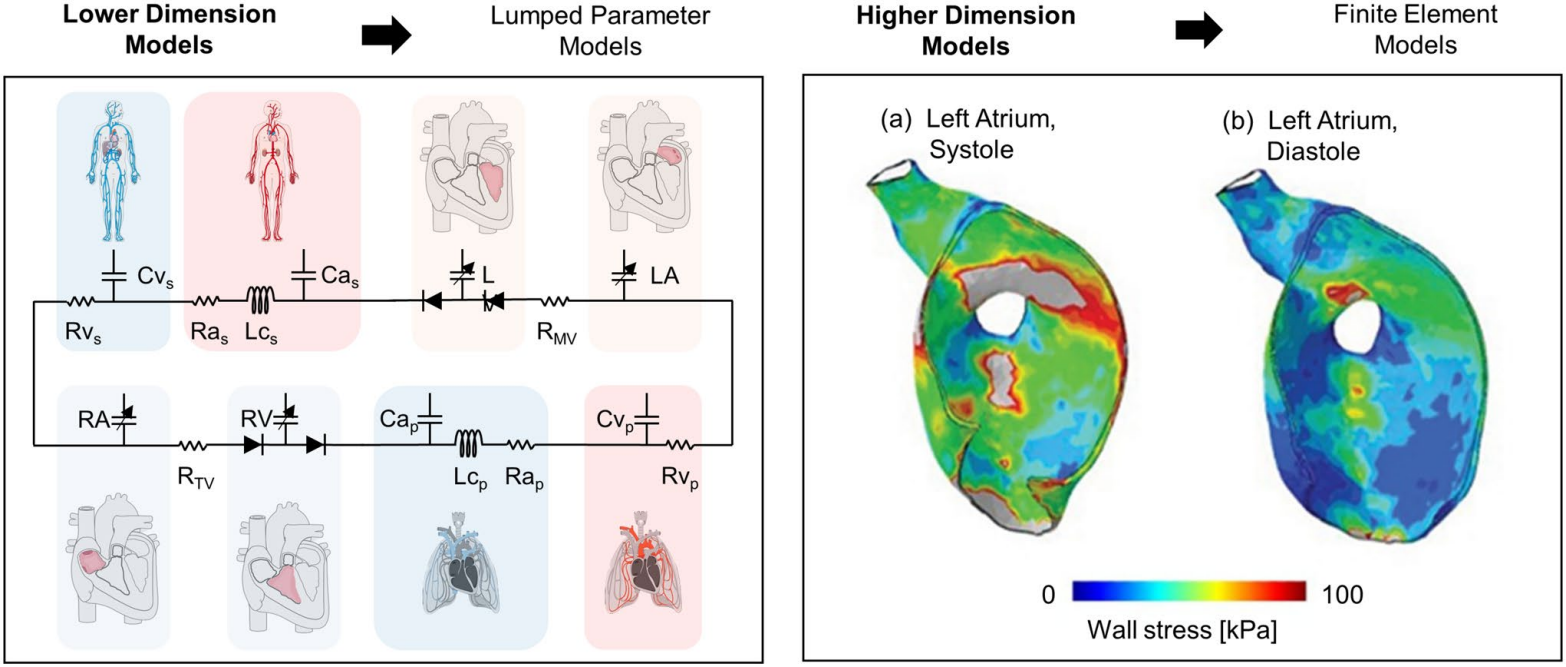
Overview of in-silico models. Lumped parameter models use electrical analogues to model the cardiovascular system, where compliance (C) and resistance (R) elements represent its hydraulic properties (left). Finite element models solve equations governing spatially and temporally resolved behavior, enabling detailed analysis of cardiac tissue mechanics and hemodynamics: Cross-sectional view of a left atrium during systole (a) and diastole (b) showing myocardial wall stress in a HFpEF model, which impacts the biomechanics of the heart (right). Ra - Arterial resistance; Rv - Venous resistance; Lc - Characteristic inductance; Ca - Arterial compliance; Cv - Venous compliance; LV - Left ventricle; LA -  Left atrium; RA - Right atrium; RV - Right ventricle. Left panel based on Kaye et al. [22]. Right panel adapted from Ozturk et al. [39]. © 2022 Ozturk, Rosalia and Roche (CC BY 4.0).

In-silico HFpEF models include lower- and higher-dimensional computational frameworks, that can be combined into multiscale models to leverage their complementary strengths (Figure 2). These models are instrumental in unravelling HFpEF pathophysiology and informing device-based interventions.

#### Lower-Dimensional Models

Lumped parameter models (LPMs) have been essential tools for replicating HFpEF-specific hemodynamic characteristics, such as elevated left atrial pressure and reduced ventricular compliance. These models provide valuable insights into systemic hemodynamics and have been widely applied to evaluate device-based therapies for HFpEF. By simplifying cardiovascular dynamics through electrical analogs, LPMs have enabled researchers to analyze the interactions between arterial, venous, and ventricular systems efficiently.

Studies utilizing LPMs have significantly contributed to understanding how HFpEF patients respond to different therapeutic devices. For instance, inter-atrial shunt devices were shown to reduce left atrial pressure for HFpEF patients [27], offering a potential intervention to alleviate HFpEF symptoms [11, 20]. Similarly, the use of LPMs has clarified the conditions under which mechanical circulatory support (MCS) devices seem most effective. A model developed by Colacino et al. [40] was adapted by Moscato et al. [29] to represent HFpEF conditions and utilized to assess the hemodynamic effects of continuous flow LVADs on HFpEF patients during rest and exercise. These modifications included adapting ventricular end-diastolic, end-systolic, and relaxation properties, as well as incorporating the hemodynamic response to exercise. The resulting model demonstrated potential benefits such as unloading of the left ventricular and pulmonary venous circulation, and increased cardiac output. Burkhoff et al. [11] evaluated the suitability of a left atrial assist device across four distinct HFpEF phenotypes, demonstrating the importance of patient-specific considerations in optimizing device deployment strategies. Granegger et al. [23] extended this work by simulating the impact of four device-based therapies under rest and exercise conditions, highlighting the dynamic nature of HFpEF and its implications for therapy development. Arduini et al. [24] illustrated how a soft robotic extra-aortic counter pulsation device could enhance diastolic filling, showcasing a LPMs' utility in assessing novel therapeutic concepts.

Beyond device evaluation, LPMs have also been used to simulate HFpEF progression and refine our understanding of its pathophysiology. Basu et al. [41] amended a cardiorenal model presented by Yu et al. [42] to better understand the heterogeneity of HFpEF by modelling combinations of various mechanisms, including myocardial, arterial, and venous stiffness, impaired relaxation, reduced contractility, hypertension, and venous capacitance, contributing to HFpEF and their effects on remodeling. Kadry et al. [19] presented further models, providing insights into how left ventricular and left atrial properties evolve with disease severity and impact therapeutic outcomes by combining a 1D arterial network and a 0D four-chamber heart simulating three HFpEF phenotypes based on myocardial relaxation delay and passive left ventricular stiffness. Kaye et al. [20] identified the role of stressed blood volume at rest and during exercise as a key factor in HFpEF pathophysiology, using a LPM informed by patient data from both HFpEF and control groups. CircAdapt and Harvi, interactive cardiovascular simulators based on LPM frameworks, have demonstrated the value of patient-specific modelling by enabling real-time simulations of HFpEF dynamics and facilitating phenotype-specific stratification for device testing [21].

However, LPMs are inherently limited in their ability to provide spatially resolved insights such as localized flow dynamics, mechanical stresses, or tissue-device interactions. For example, while these models can simulate global hemodynamics, they cannot evaluate thrombogenesis risks or predict how devices affect local tissue structures. Furthermore, LPMs rely on assumptions and simplifications that may not fully capture the complexity of HFpEF, such as its remodeling processes or interactions between the heart and other organs.

#### Higher-Dimensional Models

Higher-dimensional models, like finite element models (FEMs) and finite volume models (FVMs), offer spatial resolution, enabling detailed analyses of HFpEF-related phenomena like myocardial stress distribution, ventricular wall strain, and blood flow patterns. Accordingly, these models are essential for studying the structural and hemodynamic changes that define HFpEF, particularly its remodeling processes and responses to device support to aid device development and evaluation.

One key contribution of FEM models is their ability to simulate long-term cardiac remodeling in HFpEF. Genet et al. [25] introduced a growth model that connects sarcomere-level processes, parallel and serial sarcomere deposition during transverse and longitudinal growth, respectively, with macroscopic ventricular remodeling. The model, based on human MRI data, uses stretch-driven growth kinetics with a normalized time variable to represent remodeling over months to years, and activates growth only above physiological fiber stretch. While this approach simplifies molecular-to-organ time scale translation, it captures the cumulative impact of chronic loading and provides a framework currently being calibrated using porcine models of concentric and eccentric hypertrophy. Such models enable prediction of long-term changes in chamber size, wall thickness, and geometry, offering insights into remodeling mechanisms and could be advanced to inform device development to counteract adverse remodeling.

Weissman et al. [26] used FEM to assess structural remodeling in a porcine model of HFpEF. Cardiac MRI and pressure data were collected before and after HFpEF induction via pressure overload, and FEMs were generated via mesh morphing of the living heart porcine model. Material properties were calibrated to match passive and active myocardial behavior. The study found predominantly isotropic changes in passive properties, with myocardial thickening preserving tissue incompressibility. This method demonstrated how structural remodeling, such as alterations in left ventricular geometry, correlates with functional impairments, highlighting the importance of tailoring therapies to individual patients.

While higher-dimensional models excel in capturing localized structural and flow dynamics, they also face limitations. Their computational intensity can restrict scalability and real-time simulation capabilities. These models often lack integration with systemic hemodynamics, limiting their ability to assess how localized changes impact global cardiovascular function. These shortcomings highlight the need for coupling higher-dimensional models with LPMs to bridge the gap between local and systemic analyses.

#### Lower- and Higher-Dimensional Model Coupling

Multiscale models, combining LPMs, FEMs, and FVMs provide a comprehensive understanding of HFpEF by integrating systemic hemodynamics with localized flow dynamics and structural remodeling. These models leverage the strengths of both approaches, offering a detailed and systemic perspective on cardiovascular function [43].

Rosalia et al. [43] demonstrated the potential of multiscale modelling by coupling an LPM with a three-dimensional FEM to simulate HFpEF under conditions of aortic stenosis-induced pressure overload. This model incorporated variable-compliance chamber elements, dynamically adjusting left ventricular diastolic compliance to replicate HFpEF-specific contractility. By illustrating differences in von-Mises stress distribution in the left ventricular wall during systole and diastole for healthy and HFpEF hearts (Figure 2, right), the model highlighted the mechanical stresses contributing to disease progression. Building upon this, Ozturk et al. [39] expanded the model’s application to the design of a pulsatile MCS device. By integrating LPMs, FEMs, and FVMs, the study optimized the device for left atrial pressure reduction, a key characteristic of HFpEF. The findings suggest that pulsatile flow support not only reduces left atrial and ventricular pressures and wall stresses more effectively than continuous flow but also achieves more physiological arterial hemodynamics.

Weissman et al. [44] extended the capabilities of multiscale models by integrating MRI-derived cardiac geometries into a combined LPM-FEM framework. This approach enabled simulation of phenotype-specific HFpEF characteristics, including left ventricular hypertrophy and reduced chamber volume. By linking structural remodeling with functional capacity, the study demonstrated the importance of understanding the heterogeneity across different HFpEF phenotypes for device development and performance evaluation and may aid for device assessment under patient specific conditions in future studies.

While multiscale computational models improve upon standalone in-silico models by integrating global and local phenomena, they remain limited by their reliance on assumptions about biological systems and their inability to directly assess physical device performance. These assumptions include simplified or idealized representations of blood properties, cardiac wall motion, valve and chamber geometry, and boundary conditions, as well as numerical simplifications for computational efficiency. Additionally, while these models can simulate device-tissue interactions under controlled conditions, they cannot fully capture the complexity and variability of real-world device behavior in biological systems. To address these gaps, in-vitro models provide a physical platform to experimentally evaluate HFpEF hemodynamics and device interactions, complementing insights from computational simulations. All presented in-silico models are summarized in the Appendix, Table 1.

### In-Vitro Models

**Fig. 3:**
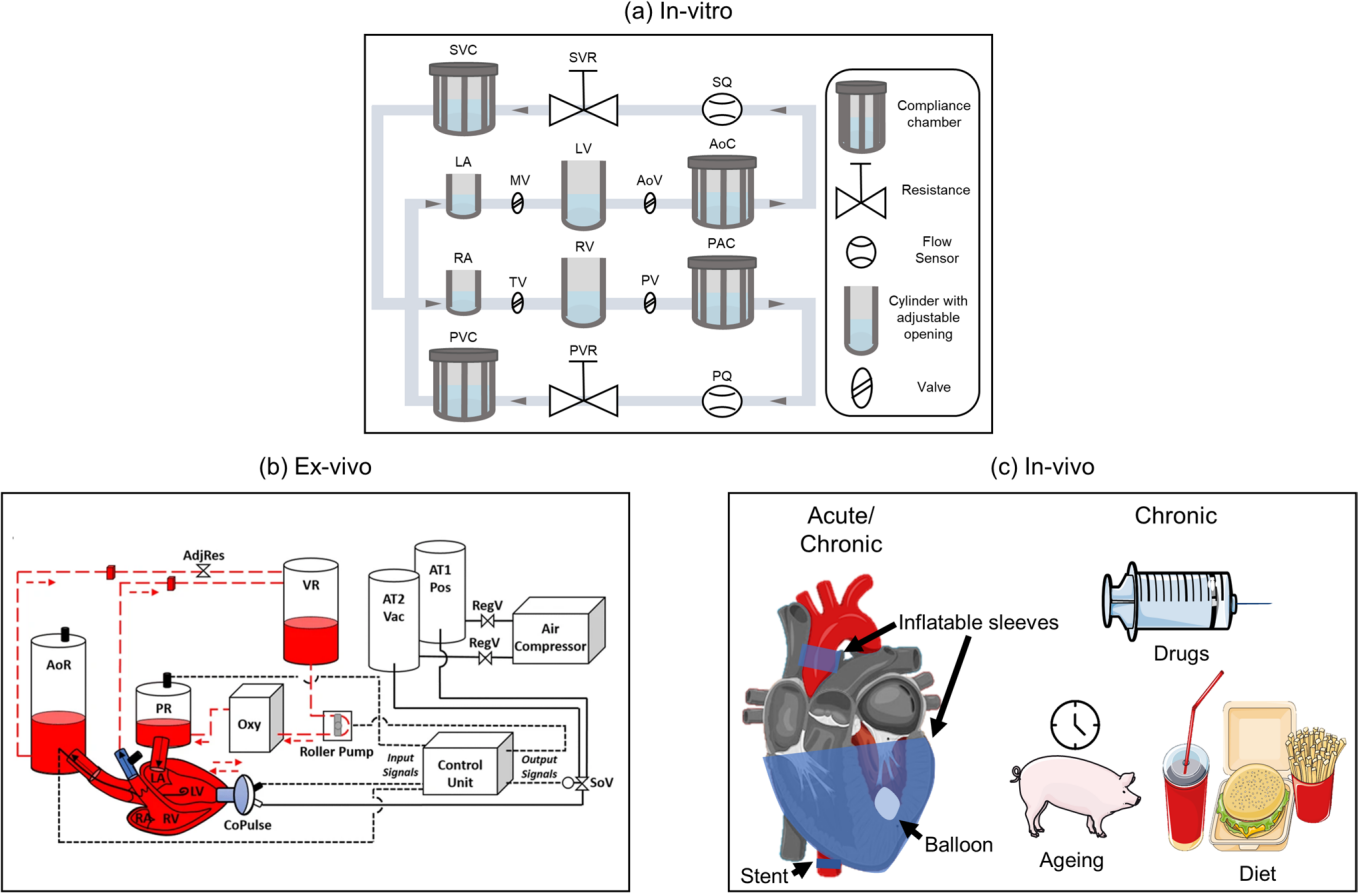
Overview of experimental HFpEF models. (a): Schematic of an in-vitro mock circulation loop (MCL) (b): Ex-vivo MCL incorporating an animal heart presented by Escher et al. [34]. (c): In-vivo models illustrating acute and chronic set-ups using mechanical devices, drugs, aging, or dietary interventions: SVC/PVC - Systemic/Pulmonary Venous Compliance; SVR/PVR - Systemic/Pulmonary Venous Resistance; SQ/PQ - Systemic/Pulmonary Flow Sensor; LA/RA - Left/Right Atrium; MV/TV - Mitral/Tricuspid Valve; LV/RV - Left/Right Ventricle; AoV/PV - Aortic/Pulmonary Valve; AoC/PAC - Aortic/ Pulmonary Artery Compliance; AoR - Aortic Reservoir; PR - Preload Reservoir; AdjRes - Adjustable Resistance clamp; Oxy - Oxygenator; VR - Venous Reservoir; AT2 Vac - Air Tank 2 for vacuum pressure; AT1 Pos - Air Tank 1 for positive pressure; RegV - Regulator Valve; SoV - Solenoid Valve; LA - Left Atrium; LV - Left Ventricle; RA - Right Atrium; RV - Right Ventricle. (a): based on Gregory et al. [45], (b): adapted from Escher et al. [34]. © 2022 Escher et al. (CC BY 4.0).

In-vitro HFpEF models, particularly MCLs, (Figure 3 (a)), provide valuable insights into the hemodynamics of HFpEF, enabling researchers to replicate and study flow rates, pressures, and volumes under controlled conditions. These models address limitations of in-silico approaches by physically representing cardiovascular dynamics and offering platforms for device testing. They are particularly useful in evaluating how physical devices influence HFpEF-specific hemodynamic characteristics, such as elevated left atrial pressures, reduced ventricular compliance, and systemic resistance.

Many MCL designs focus on the left ventricle [30, 31] or both the left ventricle and atrium [32, 33], using compliance chambers and vascular resistances to mimic cardiac and arterial dynamics. Some setups, like Langer et al. [27], include arterial and venous elements to simulate a wider range of HFpEF conditions. These models offer control over vascular resistance to reproduce different pressure states. While some use rigid PVC chambers [27, 32], others adopt patient-specific geometries from CT or MRI for greater anatomical accuracy [31, 33]. Ventricular contraction is typically driven pneumatically, via compliance chambers [27, 32, 33] or soft actuators [31], and most use blood-mimicking fluids to simulate realistic hemodynamics [27, 30]–[33].

MCLs have been used to evaluate devices such as the left atrial assist device [32], transcatheter aortic valves (Evolut R, Medtronic, Minneapolis, MN and SAPIEN 3, Edwards Lifesciences, Irvine, CA) [31], and the HeartMate 3 [27], providing critical data on their effects on HFpEF-specific hemodynamics. These set-ups have enabled studies across varying HFpEF severity levels, including mild, moderate, and severe phenotypes [32], as well as specific conditions like hypertension [30] and exercise [27], helping to identify the patient subgroups most likely to benefit from specific interventions.

Hybrid models, which combine MCLs with LPMs, further expand the utility of in-vitro systems. These models introduce time-varying boundary conditions and mimic autoregulatory responses, enabling the exploration of parameters typically not physically modelled in the MCL. For instance, Broda et al. [30] tested the HeartWare ventricular assist device (HVAD) System (Medtronic, Dublin, Ireland) in simulated patients with and without pulmonary hypertension in two configurations: from the left ventricle to the aorta and from the left atrium to the aorta. Both configurations increased cardiac output and reduced left atrial pressure; however, only left ventricular support effectively unloaded the ventricle, while left atrial decompression did not reduce left ventricular volume.

Escher et al. [34] examined the CoPulse system, a valveless pulsatile pump connected to the left ventricle designed to increase left ventricular capacity of HFpEF patients. Device support resulted in reductions in left atrial pressure and increases in cardiac output.

He et al. [28] studied the HeartMate 3, a left ventricular assist device for HFrEF patients (Abbott Laboratories, Abbott Park, Illinios, USA) in left atrial to aorta configuration. This study evaluated left atrial decompression at rest and exercise in a simulated HFpEF patient with low cardiac output, suggesting a minimum pump speed to avoid backflow and achieve left atrial decompression.

Rocchi et al. [35] advanced hybrid modelling by developing a simulator with a soft robotic patient-specific left ventricle modelling intracardiac pressure and volume waveforms, which can be applied to capture systemic responses to HFpEF therapies.

While MCLs have advanced our understanding of HFpEF hemodynamics and facilitated device testing, they have limitations. They cannot replicate biological processes, such as myocardial remodeling, or assess blood-device interactions and hemocompatibility under fully realistic conditions. Furthermore, these models lack biological tissues, limiting their utility for studying tissue-device interactions or long-term adaptive responses. All presented in-vitro models are summarized in the Appendix, Table 2.

These gaps highlight the need for models incorporating biological components, such as ex-vivo set-ups, to address tissue-specific questions and complement the insights gained from MCL studies.

### Ex-Vivo Models

Ex-vivo models (Figure 3 (b)) provide a valuable intermediary step between in-vitro systems and in-vivo studies by integrating biological components into mechanical set-ups. These models are particularly effective for studying device-tissue interactions and hemocompatibility of devices, enabling researchers to assess how cardiovascular devices influence cardiac mechanics and energetics under controlled experimental conditions. By incorporating excised biological tissues, ex-vivo set-ups allow for realistic evaluations of device performance in HFpEF-relevant scenarios.

For instance, Escher et al. [34] employed an ex-vivo model using healthy, isolated beating porcine hearts. Despite the animals being preoperatively healthy, post-experimental assessments revealed reduced diastolic compliance, consistent with pathophysiological characteristics of HFpEF. The hearts were connected to a MCL to evaluate a pump intended for HFpEF treatment (Figure 3 (b)). This approach offered critical insights into the mechano-energetic effects of the device on the left ventricle, revealing how pump support influenced cardiac function in real-time. The set-up included a dedicated blood circulation loop, with components such as a pressure-controlled preload reservoir, an aortic reservoir, a venous reservoir, an oxygenator, and a controllable roller pump. This configuration replicated physiological pressures and flows, allowing precise assessments of pump hemodynamics and its mechanical interaction with the heart. Pump support increased total cardiac output at constant left atrial pressure and resulted in higher end-systolic volumes. When cardiac output was held constant, the pump reduced left atrial pressure. Efficiency was assessed based on the pump’s ability to improve hemodynamics relevant to HFpEF, particularly by increasing cardiac output and lowering left atrial pressure. Flick et al. (2023) [46] assessed the hemocompatibility of the left atrial assist device through benchtop hemolysis testing, using bovine blood in a temperature-controlled loop. By comparing the results with those of existing blood pumps on the market, the tests confirmed that the device's normalized index of hemolysis remained within an acceptable range.

Ex-vivo models have contributed significantly to advancing our understanding of HFpEF-specific device performance by offering direct observation of acute changes in the heart to mechanical support. However, these set-ups are limited to short-term studies and cannot replicate chronic disease progression or long-term adaptive responses, such as fibrosis or ventricular remodeling, which are critical aspects of HFpEF pathophysiology. Furthermore, hemocompatibility studies in MCLs are often simplified, with static or idealized flow conditions that fail to capture the complexity of HFpEF-specific blood trauma. Moreover, the reliance on excised tissue or blood restricts the scope of phenotypes that can be studied and complicates efforts to model the systemic effects of device interventions. All presented ex-vivo models are summarized in the Appendix, Table 3.

While ex-vivo models provide unique advantages in studying device-organ interactions and hemocompatibility, their limitations highlight the importance of complementing these findings with in-vivo studies, which can address the dynamic, systemic, and long-term responses that ex-vivo set-ups cannot replicate.

### In-Vivo Models

In-vivo HFpEF models (Figure 3 (c)) are essential for exploring the disease's pathophysiology and validating device-based therapies in realistic physiological settings. They play a critical role in replicating hallmark features of HFpEF, including increased atrial pressure, diminished ventricular compliance, and progressive cardiac remodeling. They also meet essential regulatory requirements by providing critical data on safety, efficacy, and long-term impacts, bridging the gap between preclinical studies and clinical trials.

Acute in-vivo models have provided insights into the immediate hemodynamic and mechanical impacts of HFpEF interventions. For instance, Miyagi et al. [47] developed a balloon inflation model in calves to reduce left ventricular compliance and volume, effectively simulating the stiffness characteristic of HFpEF. This model quantified how left ventricular stiffness alters pressure-volume relationships and impacts ventricular filling dynamics, which are key considerations for device design. Similarly, Rosalia et al. [36] demonstrated the feasibility of using soft robotic sleeves on the aorta and the epicardium to replicate HFpEF-like pressure overload. This set-up revealed the efficacy of interatrial shunts in reducing left atrial pressure, emphasizing their potential to address elevated atrial pressures in HFpEF patients. However, acute models are limited by their short duration, failing to capture long-term adaptive mechanisms or remodeling processes. Furthermore, the risk of premature mortality and physiological instability in these set-ups underline the challenges of maintaining reproducibility and reliability.

Some chronic in-vivo models, by contrast, offer valuable insights into the progressive nature of HFpEF and its structural remodeling. Techniques like aortic banding [48]–[50] and aortic stents [51] have been instrumental in mimicking diastolic dysfunction through sustained increases in mean arterial pressure, left ventricular hypertrophy, and fibrosis. These studies have deepened our understanding of how prolonged pressure overload induces ventricular stiffness and remodeling, key pathological drivers of HFpEF. Pharmacological models, such as those utilizing deoxycorticosterone acetate, have also been used to replicate HFpEF characteristics including left ventricular hypertrophy, atrial enlargement, and tissue stiffening [52, 53]. These models have provided valuable insights on long-term structural changes, informing the design of device-based treatments targeting diastolic dysfunction and providing a platform for long-term device evaluation. However, chronic models face challenges in accurately reproducing the heterogeneous phenotypes of HFpEF, which can limit their translational applicability. Issues such as variable disease progression and premature mortality further complicate their use in preclinical research.

In-vivo models have advanced our understanding of HFpEF’s systemic impacts, including neurohormonal responses, multi-organ interactions, and autoregulatory mechanisms. Unlike in-vitro or ex-vivo set-ups, they offer the unique advantage of studying chronic device effects on remodeling processes and overall cardiovascular function. However, their inherent complexity, high cost, and ethical considerations restrict their use to later stages of the device development pipeline. Dedicated reviews [37, 38] provide a broader analysis of these models and their limitations, suggesting the need for further refinement to better replicate HFpEF heterogeneity. All presented in-vivo models are summarized in the Appendix, Table 4.

## Emerging Developments in HFpEF Modelling

**Fig. 4:**
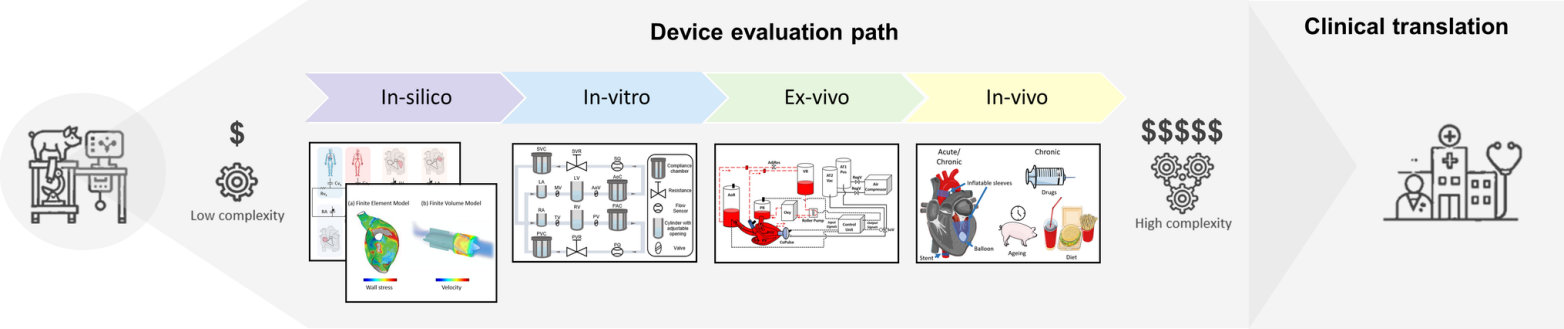
Schematic illustration of HFpEF model types used throughout the progressive device evaluation pathway. Contains graphics created with AI. In-silico are based on Kaye et al. [22] and adapted from Ozturk et al. [39]. In vitro graphic based on Gregory et al. [45]. Ex-vivo graphic adapted from Escher et al. [34]. © 2022 Ozturk, Rosalia and Roche (CC BY 4.0). © 2022 Escher et al. (CC BY 4.0).

The suite of available HFpEF models has substantially advanced our understanding of HFpEF physiology, and assisted in the early development of device-based therapies to support HFpEF patients. To date, the design and evaluation of device-based interventions for HFpEF follows a classic medical device development pipeline starting with low-cost and rapid in-silico models to inform device design and expedite initial examinations, followed by device manufacturing and evaluation using in-vitro models, before moving to time and cost intense ex-vivo and in-vivo examinations (Figure 4).

Building on this pipeline, recent technological advancements offer opportunities to overcome the limitations of current HFpEF models, paving the way for more accurate, scalable, and efficient approaches to device development. In the following section, we explore recent technological advancements that offer promising avenues to address the gaps in current HFpEF models identified in the previous section. These innovations aim to enhance the fidelity, scalability, and predictive power of in-silico, in-vitro, ex-vivo, and in-vivo models, thereby accelerating the development of device-based therapies for HFpEF.

Emerging tools emphasize the importance of tailoring interventions to individual patients, reflecting the heterogeneity of HFpEF. Integration of AI and digital twin technologies promise virtual trials that could reduce reliance on experimental models and animal studies, offering unmatched controllability and inclusivity for underrepresented groups, as well as personalized treatment [54, 55]. While virtual clinical trials for imaging [56] and drug testing [57]–[59] have been explored over the past years, the use of virtual patients for device testing is an emerging field with significant potential for growth and advancement. AI-driven virtual trials could allow researchers to conduct in silico trials that improve the design, development, testing, and monitoring of new medical devices, for example by optimizing anatomical fit [60] and evaluating hemodynamic responses in a virtual environment [61]. These simulations could also be used to investigate the potential benefits of device-based interventions in a carefully selected HFpEF population, accounting for the condition’s wide range of phenotypes. This approach could help determine whether a single device can serve all phenotypes or if different solutions are required for different subgroups. In addition, virtual trials may allow researchers to explore optimal timing for device implantation or explantation and to refine clinical protocols ahead of first in human studies.

In-vitro set-ups, such as MCLs, remain essential for physical testing of manufactured devices. Innovations like hardware-in-the-loop systems not only allow for real-time computational simulations of complex biological responses while replicating realistic, patient-specific hemodynamic conditions on the benchtop, but also facilitated device hemocompatibility testing under realistic dynamic boundary conditions [62]. This is particularly valuable for evaluating how devices behave across a range of HFpEF phenotypes, where subtle differences in preload, afterload, and ventricular stiffness can greatly impact performance. Advanced materials and additive manufacturing enable realistic environments with tunable material properties for testing device interaction with anatomically accurate geometries as well as cardiac tissue [63]–[65]. Soft robotic actuators replicate biomimetic cardiac motions, like torsion and localized wall motion abnormalities, also contributing to enhanced fidelity of in vitro models and device assessment. These enhancements increase the fidelity of device–patient interaction studies enhancing the investigation of device fitting, obstruction and efficacy under realistic and time-varying conditions. Such systems aid bridging the gap between computational predictions and physical testing, expediting design iterations.

Ex-vivo models are uniquely suited for investigating device-tissue interactions, yet they often lack the specificity required to replicate HFpEF remodeling. Emerging technologies, such as 3D bioprinting [66], enable the fabrication of engineered cardiac tissues with spatially controlled cell distribution and specific mechanical properties, mimicking disease-specific alterations such as regional fibrosis or hypertrophy. Organ-on-a-chip systems [67] can integrate human-derived cardiomyocytes and endothelial cells under controlled flow and mechanical loading conditions, facilitating the study of tissue-level responses to device implantation in phenotype-specific environments. These methods enable more precise assessments of device performance. Additionally, high-resolution imaging techniques like micro-CT and MRI [68, 69] could improve characterization of structural and functional changes in these set-ups and facilitate monitoring disease progression, device integration, and therapy response.

In-vivo models, while resource-intensive, remain critical for capturing systemic responses and long-term remodeling. Advances in genetic engineering, such as CRISPR/Cas9, could enable the creation of HFpEF-specific mutations [70, 71], allowing for more accurate replication of disease phenotypes. Similarly, imaging technologies like 4D flow MRI could provide real-time monitoring of blood flow dynamics, ventricular motion, and structural changes. This allows for detailed tracking of disease progression and device impact over time, including subtle improvements in diastolic function or flow redistribution that might be missed with traditional echocardiography. When combined with genetic models, such imaging tools can uncover how specific pathophysiological traits influence response to intervention, guiding both device optimization and patient selection strategies.

**Fig. 5:**
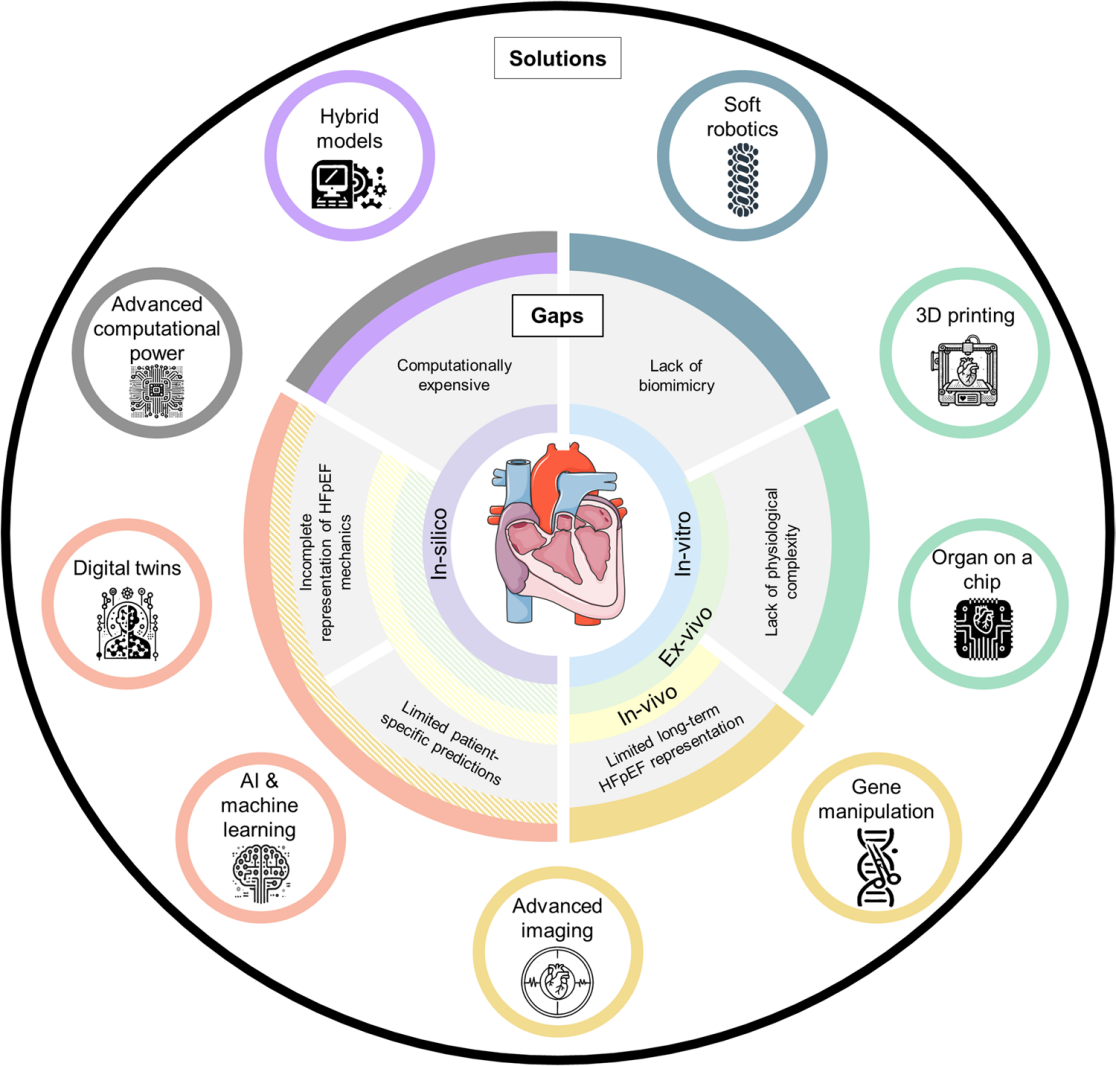
Diagram illustrating current limitations in HFpEF models (center box) and proposed tools to address these gaps (top box). Solutions are color-coded to indicate which gaps they target, and pattern-coded to show the specific models they could improve. Contains graphics created with AI.

Collectively, these innovations are transforming the landscape of HFpEF research and device development (Figure 5). By addressing the gaps in current modelling strategies, they hold the potential to accelerate the regulatory approval processes and the time-to-market for innovative therapies, ultimately improving outcomes for HFpEF patients.

## Conclusion

Pre-clinical HFpEF models are indispensable for advancing device-based treatment options, offering valuable insights into HFpEF physiology and guiding device design, validation, and testing. Each modelling strategy, whether in-silico, in-vitro, ex-vivo, or in-vivo, presents unique strengths and limitations. Emerging tools, such as advanced computational techniques, biomimetic technologies, and imaging innovations, show promise in addressing current limitations and enhancing model fidelity. Despite these advances, no single model or combination fully replicates the intricate physiology and hemodynamics of HFpEF. A comprehensive approach that integrates multiple models across different stages of development provides the most robust framework for optimizing and validating HFpEF therapies, leveraging the complementary strengths of diverse models to ensure thorough and accurate device evaluation.

## Appendix

See Tables 1, 2, 3, and 4.

**Table 1:** In-silico HFpEF models mentioned in this review sorted by method, listed with type, title, author, year, methods and results

Type	Title	Author	Year	Methods	Results
Lower dimensional model	Effects of an interatrial shunt on rest and exercise hemodynamics: results of a computer simulation in heart failure	Kaye et al. [22]	2014	An LPM simulated rest and exercise hemodynamics in HFpEF using data from two prior studies to evaluate the theoretical effects of an interatrial shunt (up to 12 mm diameter).	The shunt reduced PCWP by ~ 3 mm Hg at rest and ~ 11 mm Hg during exercise, with consistent left-to-right flow; most of the effect was achieved with an 8-9 mm shunt. Left ventricular output decreased slightly, while right ventricular output increased accordingly, without raising right atrial or pulmonary artery pressures.
	Use of continuous flow ventricular assist devices in patients with heart failure and a normal ejection fraction: a computer-simulation study	Moscato et al. [29]	2013	A previously validated cardiovascular model [40] was adapted to simulate HFpEF hemodynamics using published patient data. A continuous-flow LVAD was incorporated to assess its hemodynamic effects at rest and during exercise.	The model reproduced patient hemodynamics within clinical variability. LVAD support reduced LV end-diastolic pressure and volume at rest and during exercise, lowered left atrial pressure, and slightly increased cardiac output during exercise.
	Left atrial decompression pump for severe heart failure with preserved ejection fraction: theoretical and clinical considerations	Burkhoff et al. [11]	2015	Assess a low-flow micropump-based LA decompression device, comparing inflow from the left atrium versus the left ventricle using LPM tuned to 4 phenotypes: 1) hypertrophic cardiomyopathies; 2) infiltrative diseases; 3) nonhypertrophic HFpEF; 4) HFpEF with common cardiovascular comorbidities.	MCS improved cardiac output, modestly increased blood pressure, and reduced pulmonary and left atrial pressures across all HFpEF phenotypes. LV sourcing risked suction due to reduced end-systolic volume, whereas LA sourcing increased LV end systolic volumes and minimized suction risk due to pre-existing LA enlargement.
	Comprehensive Physiological Modeling Provides Novel Insights Into Heart Failure With Preserved Ejection Fraction Physiology	Kaye et al. [20]	2021	Simultaneous right-heart catheterization and echocardiography were performed at rest and during exercise in 60 HFpEF patients and 22 healthy controls. Individual patient-level hemodynamic and LV function data were used in simulations to estimate circulatory parameters contributing to pulmonary capillary pressure, including stressed blood volume.	HFpEF patients had higher LV stiffness, elastance, and stressed blood volume than controls at rest and during exercise. During exercise, increased stressed blood volume and LV stiffness were strongly associated with elevated pulmonary capillary wedge pressure. Simulations revealed that the hemodynamic response to exercise arises from complex nonlinear interactions between circulatory parameters.
	From diastolic dysfunction to exercise intolerance: an in silico simulation study on the phenotypic markers of heart failure with preserved ejection fraction	Van Loon et al. [21]	2020	Using the CircAdapt model, simulations assessed the effects of impaired LV relaxation and increased myocardial stiffness on hemodynamics, based on a hypertensive reference state with concentric hypertrophy. LV stiffness and relaxation were varied, and outputs included LVEF, E/A ratio, mean LA pressure, and maximum cardiac output during exercise.	Despite preserved LVEF, impaired relaxation and increased LV stiffness led to abnormal filling patterns, elevated mean LA pressure, and reduced exercise capacity. COmax declined by 15-53% depending on severity and LA involvement, highlighting the combined impact of LV and LA dysfunction on hemodynamics.
	Biomechanics of diastolic dysfunction: a one-dimensional computational modeling approach	Kadry et al. [19]	2020	A computational model combining a 1D arterial network and a 0D four-chamber heart was developed to study diastolic dysfunction. Parameter sensitivity analysis was performed on myocardial relaxation delay (early and late phase) and passive LV stiffness, and all parameters were combined to simulate three diastolic dysfunction phenotypes.	The model successfully reproduced impaired relaxation, pseudo-normal, and restrictive phenotypes of diastolic dysfunction by selectively adjusting relaxation delay and passive stiffness. The study provided biomechanical insight into how these parameters shape the development and manifestation of diastolic dysfunction.
	Comparison of device-based therapy options for heart failure with preserved ejection fraction: a simulation study	Granegger et al. [23]	2022	A lumped parameter cardiovascular model was used to simulate rest and exercise hemodynamics in two HFpEF subgroups and to evaluate the effects of four device-based therapies: an interatrial shunt, LV-VAD, LA-VAD, and the CoPulse valveless pulsatile assist device.	All four devices reduced left atrial pressure by > 20% at rest and during exercise. IASDs lowered cardiac output and increased pulmonary load, while mechanical assist devices increased output and reduced sympathetic activity. LV-VADs posed suction risk. Findings support phenotype-specific, individualized therapy selection in HFpEF.
	Framework for patient-specific simulation of hemodynamics in heart failure with counterpulsation support	Arduini et al. [24]	2022	A patient-specific lumped parameter model of HFpEF was developed and validated against clinical imaging data to evaluate a soft robotic extra-aortic counterpulsation device, modelled as a pressure source and capacitance. The device was experimentally characterized and tested in silico to assess the impact of actuation timing on hemodynamics.	The model closely matched patient data, with < 5% error in most parameters. Actuation 350 ms before systole reduced systolic pressure and stroke work, slightly increased cardiac output and coronary flow, but led to a rise in LA pressure. Higher actuation pressure was associated with greater systolic pressure reduction and modestly increased coronary flow.
	Understanding heterogeneous mechanisms of heart failure with preserved ejection fraction through cardiorenal mathematical modeling	Basu et al. [41]	2023	A previously developed cardiorenal model [42] was used to assess the sensitivity of LV end-diastolic pressure to various mechanisms contributing to HFpEF and HFrEF, including myocardial, arterial, and venous stiffness, impaired relaxation, reduced contractility, hypertension, and venous capacitance. The effects of these mechanisms on cardiac remodeling were then assessed under both stress-driven and strain-driven assumptions to explore their impact on EF stability.	LV stiffness was the most sensitive factor influencing elevated LVEDP. Simulations showed that preserved EF in HFpEF can result from reduced strain-driven remodeling in a stiff myocardium. The model highlights how various combinations of impairments can lead to HFpEF heterogeneity and provides a mechanistic distinction from HFrEF.
Higher dimensional model	Modeling Pathologies of Diastolic and Systolic Heart Failure	Genet et al. [25]	2016	A multiscale computational model was developed to simulate individualized cardiac growth and remodeling in chronic heart failure by linking molecular sarcomere deposition processes with cellular and whole-organ mechanics.	The model predicted changes in wall thickness, chamber size, and geometry consistent with clinical observations. Unlike simpler models, it also captured secondary effects such as papillary muscle displacement, annular dilation, regurgitation, and outflow obstruction, supporting its potential for personalized treatment planning.
	Material property alterations for phenotypes of heart failure with preserved ejection fraction: A numerical study of subject-specific porcine models	Weissmann et al. [26]	2022	Two healthy swine underwent progressive pressure overload to induce HFpEF-like characteristics. cMRI and intracardiac pressures were recorded before and after induction, and finite element models were created via mesh morphing of the Living Heart Porcine model and calibrated to passive and active myocardial behavior.	The passive tissue response was predominantly isotropic, and myocardial thickening enabled a smooth mechanical transition while preserving incompressibility. The results underscore hypertrophy as an early compensatory response in diastolic heart failure.
	Lumped-Parameter and Finite Element Modeling of Heart Failure with Preserved Ejection Fraction	Rosalia et al. [43]	2021	Two computational models were developed to simulate HFpEF due to LV pressure overload: a 0D lumped-parameter Windkessel-type model and a 3D finite element model incorporating electromechanics, structural deformation, and hemodynamics. Pressure overload was simulated by reducing aortic valve area, and chronic remodeling by decreasing LV wall compliance. Follow up work includes investigation of elevated LA pressures, LA-Ao pump configuration and LV suction events under continuous and pulsatile flow support [39].	Both models reproduced hallmark HFpEF features, including increased transaortic pressure gradient, reduced stroke volume, and decreased LV end-diastolic volume. The FEA model additionally showed elevated myocardial stress throughout the cardiac cycle compared to healthy tissue. Follow-up simulations suggested that pulsatile-flow support more effectively reduced left heart pressures and wall stress while providing more physiologic arterial hemodynamics than continuous-flow support, supporting further development of pulsatile MCS devices for HFpEF [39].
	Cardiac mesh morphing method for finite element modeling of heart failure with preserved ejection fraction	Weissmann et al. [44]	2022	A reconstruction algorithm was developed to morph an existing high-resolution finite element mesh to match porcine cardiac anatomies from cMRI scans. Using iterative FE simulations with visco-hyperelastic materials, the approach preserved anatomical features such as myofiber orientation, enabling contraction and relaxation modeling for both healthy and HFpEF-induced hearts.	The algorithm successfully replicated key features of cardiac remodeling, including myocardial thickening and reduced ventricular volume. It enabled generation of subject-specific models with consistent mesh connectivity, allowing spatial comparison of pathological changes.

*LPM* lumped parameter model, *PCWP* pulmonary capillary wedge pressure, *LVAD* left ventricular assist device, *HFpEF* heart failure with preserved ejection fraction, *LV* left ventricle, *MCS* mechanical circulatory support, *LA* left atrium, *LVEF* left ventricular ejection fraction, *CO* cardiac output, *VAD* ventricular assist device, *EF* ejection fraction, *LVEDP* left ventricular end diastolic pressure, *MRI* magnetic resonance imaging, *Ao* aorta

**Table 2:** In-vitro HFpEF models mentioned in this review sorted by method, listed with type, title, author, year, methods and results

Type	Title	Author	Year	Methods	Results
MCL	Left atrial assist device for heart failure with preserved ejection fraction: initial results with torque control mode in diastolic heart failure model	Miyagi et al. [32]	2021	An in vitro MCL was used to evaluate a left atrial assist device (LAAD) across multiple timing control settings under simulated normal and diastolic heart failure conditions. The device was positioned between the left atrium and a pneumatic mock ventricle, replicating post-implantation hemodynamics.	In vitro testing showed that the LAAD restored cardiac output and aortic pressure to normal levels even under severe diastolic heart failure conditions, with minimal regurgitation. These results support the potential of the LAAD as a therapeutic option for HFpEF.
	In vitro benchtop mock circulatory loop for heart failure with preserved ejection fraction emulation	Malone et al. [33]	2022	A novel mock circulatory loop with independently actuated left atrial and ventricular chambers was developed to simulate healthy and HFpEF conditions. Two configurations, rigid and anatomically realistic soft chambers, were implemented to support evaluation of mechanical circulatory support devices for HFpEF.	Both mock loop configurations produced similar performance and successfully reproduced HFpEF-like hemodynamics, including reduced LV end-diastolic volume and elevated diastolic pressure. These responses were consistent with clinical data, demonstrating the loop’s relevance for evaluating potential HFpEF therapies.
	Soft robotic patient-specific hydrodynamic model of aortic stenosis and ventricular remodeling	Rosalia et al. [31]	2023	A soft robotic cardiovascular model incorporating 3D-printed, patient-specific anatomy and actuated sleeves was developed to replicate aortic stenosis and associated diastolic dysfunction. The system was validated against clinical data and used to assess transcatheter valve therapies across diverse patient profiles.	The model revealed that undersized transcatheter valves resulted in greater paravalvular leak and less favourable hemodynamic outcomes compared to appropriately sized implants. These findings aligned with clinical evidence linking poor sealing to increased patient risk.
	HeartMate 3 for Heart Failure with Preserved Ejection Fraction: In Vitro Hemodynamic Evaluation and Anatomical Fitting	Langer et al. [27]	2024	A cardiovascular simulator was used to evaluate a LA-Ao HeartMate3 (HM3) configuration in a simulated HFpEF patient. Hemodynamic performance was assessed at rest and during exercise across multiple pump flow rates.	Simulations showed that HM3 support in an LA-Ao configuration effectively reduced mean left atrial pressure during rest and exercise. The results suggest potential for this approach to relieve pulmonary congestion in HFpEF patients.
Hybrid MCL	Hemodynamic Improvement with Application of Mechanical Circulatory Support in Heart Failure with Preserved Ejection Fraction in a Mock Circulation Loop	Broda et al. [30]	2019	An MCL was used to simulate HFpEF with and without pulmonary hypertension by adjusting resistive and compliant elements and replicating the Frank-Starling mechanism. Hemodynamics were recorded with a HeartWare HVAD™ tested in two support configurations: LV-Ao and LA-Ao, with pump speeds set to achieve systemic flows of 4.0 and 4.5 L/min.	Both LV-Ao and LA-Ao HVAD configurations reduced LV end-diastolic pressure, LA pressure, and mean pulmonary artery pressure. LV-Ao support decreased LV volumes, improving EF and reducing stroke work and potential energy. LA-Ao increased LV volumes and potential energy but reduced stroke work.
	A Valveless Pulsatile Pump for Heart Failure with Preserved Ejection Fraction: Hemo- and Fluid Dynamic Feasibility	Escher et al. [34]	2020	An in vitro MCL was used to evaluate the hemodynamic performance of a pneumatically driven assist device (CoPulse). The device was synchronized with the LV cycle using a pressure-based control system implemented in MATLAB Simulink and deployed on a real-time control platform.	The CoPulse pump reduced LA and pulmonary artery pressures while increasing Ao pressure and cardiac output under HFpEF-like conditions. It showed favourable volumetric and work efficiency, with performance varying by HFpEF phenotype. In vitro pressure-volume loops closely matched simulation results, supporting the reliability of the experimental setup.
	Left Atrial Decompression With the HeartMate3 in Heart Failure With Preserved Ejection Fraction: Virtual Fitting and Hemodynamic Analysis	He et al. [28]	2024	A hybrid MCL was used to evaluate HM3 performance for LA decompression in HFpEF. The setup combined a physical pump, a hydraulic-pneumatic network, and a real-time control system running a cardiovascular model. Simulations of rest and exercise conditions guided pump speed adjustments to identify settings that achieved target LA pressure ranges without backflow or aortic valve closure.	In vitro testing showed that LA decompression with the HM3 normalized LA pressures without backflow, and required higher speeds during exercise compared to rest. These results support the hemodynamic feasibility of this approach for HFpEF management.
	A patient-specific echogenic soft robotic left ventricle embedded into a closed-loop cardiovascular simulator for advanced device testing	Rocchi et al. [35]	2024	A hybrid cardiovascular simulator combining a soft robotic LV and an in silico LPM was developed to replicate patient-specific HFpEF conditions. Three individual profiles were simulated to assess the system's ability to reproduce varied ventricular mechanics and hemodynamics.	The hybrid simulator successfully reproduced realistic physiological and anatomical cardiac behavior. Its combination of soft robotics and in silico modeling offers a valuable platform for device optimization, validation, and defining use-case conditions.

*MCL* mock circulation loop, *LAAD* left atrial assist device, *HFpEF* heart failure with preserved ejection fraction, *LV* left ventricle, *HM3* HeartMate 3, *LA* left atrium, *Ao* aorta

**Table 3:** Ex-vivo HFpEF models mentioned in this review sorted by method, listed with type, title, author, year, methods and results

Type	Title	Author	Year	Methods	Results
Ex-Vivo	A Valveless Pulsatile Pump for Heart Failure with Preserved Ejection Fraction: Hemo- and Fluid Dynamic Feasibility	Escher et al. [34]	2020	An isolated beating porcine heart setup was used to evaluate the interaction between a functional left ventricle and a pump prototype (CoPulse). The ex vivo model included preload and afterload control, flow and pressure sensors, and a pressure-volume catheter to assess hemodynamic response during pump support.	CoPulse support increased aortic pressure and cardiac output while reducing stroke work and raising potential energy. It effectively lowered left atrial pressure, with support conditions leading to increased end-systolic volume.
	Hemolysis Using Left Atrial Assist Device with Constant Flow: Preliminary Testing of Initial Design	Flick et al. [46]	2023	Hemolysis tests were conducted to evaluate the blood compatibility of the left atrial assist device (LAAD) in a temperature-controlled in vitro loop using bovine blood. The setup measured pump flow, inlet/outlet pressures, and hemolysis at two pump speeds. Hematocrit and plasma-free hemoglobin levels were used to calculate the normalized index of hemolysis.	The LAAD maintained consistent flow during testing and showed low levels of hemolysis across all test conditions. Its performance was comparable to previously evaluated devices and remained within acceptable limits for blood compatibility.

*LAAD* left atrial assist device.

**Table 4:** In-vivo HFpEF models mentioned in this review sorted by method, listed with type, title, author, year, methods and results

Type	Title	Author	Year	Methods	Results
Balloon inflation	Large animal models of heart failure with preserved ejection fraction	Miyagi et al. [47]	2022	A novel in vivo HFpEF model was developed in healthy calves by inserting and inflating a balloon within the LV to reduce compliance and cavity volume without disrupting contraction.	The balloon-based model successfully reproduced key hemodynamic features of diastolic dysfunction without interfering with cardiac contraction. While promising for short-term device evaluations, further development is needed to confirm its suitability for chronic studies and to distinguish diastolic from potential systolic impairment.
Robotic sleeve	Modulating Cardiac Hemodynamics Using Tunable Soft Robotic Sleeves in a Porcine Model of HFpEF Physiology for Device Testing Applications	Rosalia et al. [36]	2023	This study presents a porcine HFpEF model using implantable soft robotic sleeves on the LV and aorta to modulate compliance and afterload. The model allows controlled recreation of various HFpEF hemodynamic states and was used to evaluate the response to interatrial shunt device implantation.	The soft robotic porcine HFpEF model successfully replicated hemodynamic changes following interatrial shunt device implantation, with outcomes aligning with prior in silico and clinical findings.
Aortic bending	Natriuretic peptides in sheep with pressure overload left ventricular hypertrophy	Charles et al. [48]	1996	A chronic pressure overload model was induced in sheep using an inflatable aortic occluder. Hemodynamics, blood, and urine were monitored over six weeks, with post-mortem analysis of cardiac structure and peptide levels compared to controls.	Aortic banding in sheep induced progressive hypertension, with increased mean aortic pressure and decreased distal systolic pressure. Post-mortem analysis confirmed LV hypertrophy and cardiac remodelling. Elevated natriuretic peptides indicated heart failure progression, leading to death in most animals.
	Progressive induction of left ventricular pressure overload in a large animal model elicits myocardial remodelling and a unique matrix signature	Yarbrough et al. [49]	2012	Left ventricular pressure overload was induced in pigs through weekly ascending aortic cuff inflation over four weeks. Cardiac structure and function were assessed and compared to controls, alongside evaluation of extracellular matrix remodelling via gene expression analysis.	Pressure overload led to increased LV mass and myocardial stiffness without affecting ejection fraction. Myocardial collagen content and cross-linking rose, while gene expression of fibrillar collagens and matrix metalloproteinases remained stable. However, expression of tissue inhibitors of matrix metalloproteinases was markedly elevated.
	Myocardial ATP hydrolysis rates in vivo: a porcine model of pressure overload-induced hypertrophy	Xiong et al. [50]	2015	Aortic banding was used to induce LV hypertrophy in swine. Cardiac structure and function were assessed via MRI, and myocardial energetics were evaluated using a double-saturation phosphorous-31 magnetic resonance spectroscopy saturation transfer technique to measure ATP hydrolysis rates in vivo. Hearts were also studied under catecholamine stimulation to assess response to increased workload.	Aortic banding induced acute LV dysfunction and reduced energetic efficiency, with recovery by week 8 as hypertrophy restored wall stress. ATP hydrolysis rates increased linearly with workload (rate-pressure product) under catecholamine stimulation, in both normal and hypertrophic hearts.
Aortic stent	Porcine model of progressive cardiac hypertrophy and fibrosis with secondary postcapillary pulmonary hypertension	Gyöngyösi et al. [51]	2017	A fixed-diameter stent was implanted in the descending aorta of growing pigs to induce progressive LV pressure overload. Cardiac function and remodelling were monitored using hemodynamics, pressure-volume loops, echocardiography, and MRI. Sham-operated pigs served as controls.	Aortic stenting induced a significant pressure gradient and elevated pulmonary artery pressure, resulting in concentric LV hypertrophy and increased right ventricular mass. Histology confirmed fibrosis and myocyte hypertrophy in both ventricles. Markers of cardiac stress and dysfunction were upregulated, indicating progressive heart failure.
Pharmacological	A porcine model of hypertensive cardiomyopathy: implications for heart failure with preserved ejection fraction	Schwarzl et al. [52]	2015	A porcine HFpEF model was induced using deoxycorticosterone acetate (DOCA) and a Western diet (WD) over 12 weeks to cause hypertension and hyperlipidemia. Hemodynamic assessments were performed at rest and under stress (pacing and dobutamine). LV tissue was analysed for structural and molecular changes.	DOCA/WD pigs developed concentric LV hypertrophy, left atrial dilation, and reduced diastolic compliance with preserved ejection fraction. Under stress, cardiac reserve was impaired. Tissue analysis revealed structural and molecular changes associated with HFpEF pathophysiology.
	Early-stage heart failure with preserved ejection fraction in the pig: a cardiovascular magnetic resonance study	Reiter et al. [53]	2016	Cardiac MRI at rest and during dobutamine stress was used in DOCA and control pigs to assess cardiac structure, function, flow, and strain. Perfusion reserve was measured via coronary sinus flow, and tissue samples were analysed post-mortem for fibrosis and interstitial volume.	DOCA pigs showed increased LV mass and wall thickness. Despite preserved systolic reserve, diastolic dysfunction was evident with impaired left atrial function, reduced myocardial relaxation, and higher estimated filling pressures based on elevated E/E′. Perfusion reserve was reduced, and tissue analysis revealed increased myocardial collagen content.

*HFpEF* heart failure with preserved ejection fraction, *LV* left ventricle, *ATP* adenosine triphosphate, *DOCA* deoxycorticosterone acetate, *MRI* magnetic resonance imaging, *WD* Western diet
